# The burden of proof: The process of involving young people in research

**DOI:** 10.1111/hex.12870

**Published:** 2019-02-15

**Authors:** Gail Dovey‐Pearce, Sophie Walker, Sophie Fairgrieve, Monica Parker, Tim Rapley

**Affiliations:** ^1^ Institute of Health and Society Newcastle University Newcastle upon Tyne UK; ^2^ Northumbria Healthcare NHS Foundation Trust North Shields UK; ^3^ Department of Social Work, Education and Community Wellbeing Northumbria University Newcastle upon Tyne UK

**Keywords:** involvement, participation, patient and public involvement, research, young people, young researchers, youth research

## Abstract

Patient and public involvement in research includes non‐academics working with researchers, on activities from consultative tasks, to joint working, and on user‐led initiatives. Health and social care funding bodies require involvement in research projects. A current debate focuses on a perceived lack of empirical “proof” to demonstrate the impact of involvement upon the quality of research. It is also argued that the working relationships between researchers and those becoming involved need to be understood more fully. These areas are beginning to be reported upon but there are few studies of young people involved in health research. This study describes the experiences of adult academics and young people, working together on a large‐scale, UK health research programme. Using qualitative interview and focus group methods, the aim was to explore participants’ perceptions about the process and outcomes of their work together. The importance of cyclical, dynamic and flexible approaches is suggested. Enablers include having clear mechanisms for negotiation and facilitation, stakeholders having a vision of “the art of the possible,” and centrally, opportunities for face‐to‐face working. What is needed is a continuing discourse about the challenges and benefits of working with young people, as distinct from younger children and adults, understanding the value of this work, without young people having to somehow “prove” themselves. Involvement relies on complex social processes. This work supports the view that an improved understanding of *how* key processes are enabled, as well as *what* involvement achieves, is now needed.

## 1 INTRODUCTION

### 1.1 Overview of involvement activities

“Patient and public involvement” refers to the roles for service users and members of the public in defining, delivering and disseminating research. INVOLVE (formerly Consumers in NHS Research), an organization that supports involvement in research, was set up in 1996 in the United Kingdom by the Department of Health, to guide patient and public involvement in health and social care research.^1^

Involvement includes activities on a continuum from consultative tasks, through to “partnership working,” to service‐user‐led initiatives. Involving those with lived experience is perceived as the “right” thing to do on moral, democratic and epistemological grounds. Systematic reviews suggest that it can influence all stages of research and, in broad terms, improves the “real‐life” relevance of the work.^2^

### 1.2 “Quality” of involvement activities

One debate focuses on a perceived lack of data to demonstrate the *impact* of involvement upon research,^3^, ^4^, ^5^ with a drive to outline standards of good practice.^6^ The PIRICOM systematic review notes difficulties in achieving this:

The poor reporting of [involvement] impact and the limited consideration of how context and process factors affect impact makes meaningful comparison across studies difficult, and so prohibits firmer conclusions about their influence.^3^

Members of the PIRICOM team developed Guidance for Reporting Involvement of Patients and Public (GRIPP / GRIPP2),^7^, ^8^ calling for better reporting of practice. They echo other suggestions for a focus on *how* involvement works, as well as *what* it achieves.^9^, ^10^, ^11^ A realist evaluation study^12^ suggested that six actions support effective involvement: a shared understanding of the purposes of involvement; coordination; diversity of voices; researcher engagement; working relationships; and proactive evaluation of activities.

Importantly, involvement in health research has also been described as occupying “liminal knowledge spaces,” in between established academia and novel practice, where difference, ambiguity and tensions come to the fore, creating opportunities for transformation.^13^ Most studies focus on adults and less is understood about the experiences of young people involved in research.

### 1.3 Children's and young people's involvement

The United Nations Convention on the Rights of the Child^14^ establishes that all children have the right to be involved in decisions that affect them. Hart^15^ outlines broad approaches to the participation of children and young people, and more recently, approaches for involving them in research have been proposed.^16^, ^17^ Examples include them being involved in systematic reviews,^18^ expressing service preferences,^19^ and commissioning decisions.^20^ Guidance for involving young people and specific groups who are less frequently heard is available^6^, ^21^, ^22^, ^23^, ^24^, ^25^, ^26^ often drawing from experiences in other social science disciplines.^27^

A literature review suggested that in research *on* children, *with* children and *by* children, children's perspectives can be gained.^28^ In a case study review,^29^ it was suggested that children and young people should be involved throughout the research process, but if this is not achievable, they can still be involved in a meaningful way, with the onus on researchers to ring‐fence sections where they can collaborate and lead on tasks.

In this paper, we show that young people can meaningfully contribute to a large‐scale health research programme. We suggest that adult researchers might re‐evaluate assumptions about the capabilities of young people as researchers, without a burden upon them to prove their worth. However, this does not mean that adult involvement practices can be adopted uncritically. We need a continuing discourse about the challenges and benefits of collaboration with young people, as distinct from younger children and adults. We hope this work makes a timely contribution, by highlighting techniques and approaches that could be useful in working with those aged around 11‐25.

### 1.4 Aim

This study describes the experiences of adult researchers and young people involved in a large‐scale, UK health research programme, exploring the process of working together and the outcomes of that work.

## 2 METHODS

The “Transition” study was a 5‐year longitudinal health research programme, supported by the National Institute of Health Research, examining how health services in the UK can support young people in their move from childhood to adulthood. It comprised nine work packages, with one focusing on young people's involvement in the programme. The young people's group, formed in 2013 in the first months of the programme, met once a month to carry out their work. They called their group United Progression (UP).

When recruited, the UP group members were all aged between 15 and 20. They had experience of accessing health‐care services. Most had experience of living with physical and/or developmental conditions, many in line with the exemplar health needs being studied within the programme. Recruitment occurred in different ways (eg health services; schools; health action groups). With membership growing steadily, the group had over 20 members, with active participation fluctuating in line with examinations and other commitments. Most meetings had around eight members present.

The group's work into the “Transition” programme was facilitated by four (adult) involvement facilitators, one of whom had additional responsibilities as involvement lead, and four peer support workers (PSWs). The PSWs were recruited from a local NHS youth group, to provide input to research tasks in the first 1‐2 months, before the other young people had been recruited. They then offered initial support to newly recruited members. The PSWs became embedded members of the UP Group, as a natural part of the group's formation. This peer approach has since been reported elsewhere as useful for enabling the voices of those who tend not to participate.^16^, ^29^ The UP Group’s role was to provide a young people's perspective, with the aim of working with the adult researchers to oversee the governance and delivery of the Transition programme.^30^ The involvement lead was a member of the research team and reported to the research management meetings with the young people attending these meetings, when adults or young people felt there was a need.

**Figure 1:**
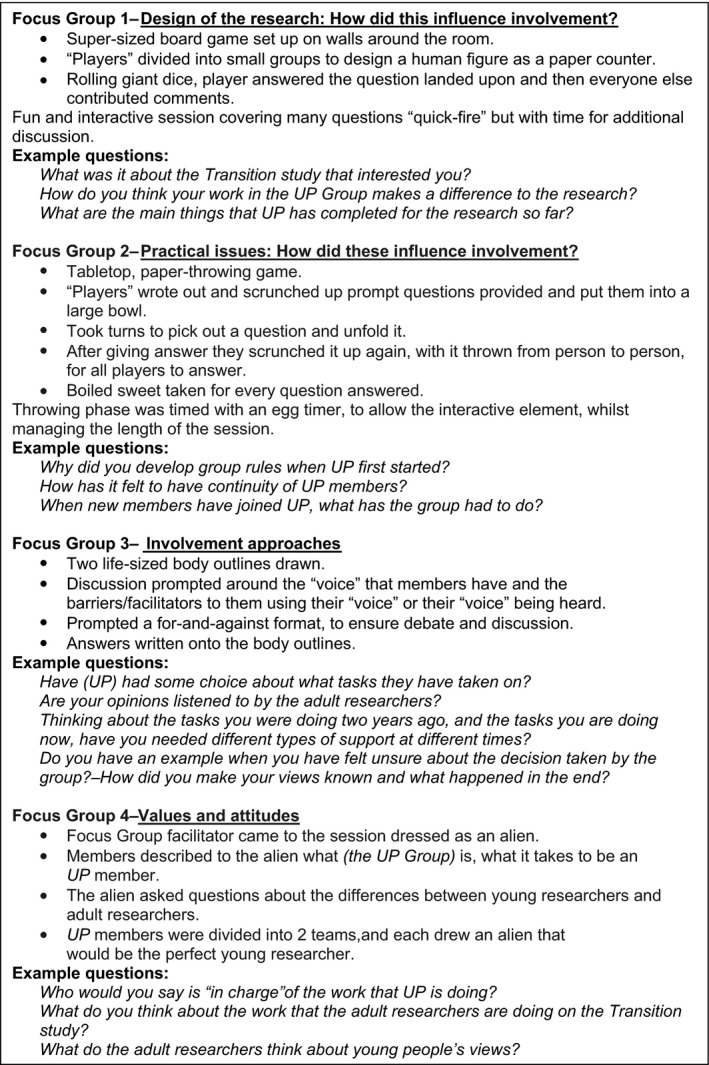
Methods for focus groups with (UP Group) members

At the initiation of this study, there were no pre‐defined standards against which to measure involvement work (two are currently being developed^31^, ^32^). Therefore, it was considered that an examination of the “process factors” (eg context; change over time; relationships), as well as more concrete outcomes, was required. Rigorous qualitative methods, carried out by members of the team with awareness of potentially significant process issues and juncture points over the years, were considered the most appropriate way to investigate the work. All authors (ie two adult researchers and three young people) collaborated to design the data collection methods. Qualitative interviews with the senior academic researchers and involvement facilitators from the Transition programme were carried out (n = 10). One person (the involvement lead) carried out the interviews. Four iterative focus groups with UP Group members took place. Six UP Group members opted in. The PSWs carried out the focus groups, with guidance from the involvement lead. They developed creative and accessible focus group methods (see Figure 1). Initial prompt questions for the interviews and focus groups were based on existing literature and developed through reflective discussions.

Interviews were recorded, anonymized and transcribed verbatim. Focus groups were transcribed in real time by two note‐keepers working independently (ie the involvement lead and one of the involvement facilitators), and the exercise outputs were captured in written and drawn materials.

The central analysis was conducted by the involvement lead (Gail Dovey‐Pearce) and a member of the research team with expertise in qualitative methods (Tim Rapley) according to the standard procedures of rigorous thematic analysis.^33^ They worked independently on sections of the data, regularly coming together for discussions of their analyses, to interrogate their coding frameworks and interpretations. Techniques from first‐generation grounded theory—coding and constant comparison^34^—were used, alongside deviant case analysis,^35^ mapping,^36^ analyst triangulation^37^ and member checking.^38^ The PSWs (Sophie Walker, Sophie Fairgrieve and Monica Parker) received periodic drafts of the analyses. They specifically engaged with the analysis of the focus group data. The UP Group received presentations of the focus group data analysis at the mid‐point and end point, to consider if themes reflected their discussions.

The paper is informed by GRIPP2^8^ reporting standards.

## 3 RESULTS

When academics and young people come together, formative cycles of work occur, as values, attitudes and practices develop. These cycles have the potential to increase perceived value and decrease doubts. There is also the potential for continuing doubt and active management needs to happen in order for the “work of involvement” to progress.

### 3.1 Hopes and doubts

The adult researchers understood the rights of young people to be involved and the importance of avoiding tokenism. They held assumptions about what they might add, in terms of bringing new knowledge and increasing accountability. Their early hopes fitted with broader moral, epistemological and democratic arguments for involvement but were often quite abstract ideals. Consequently, understanding how best to involve young people was, at times, anxiety‐provoking. For example, an adult researcher remembered a specific team meeting:

There were different views if I remember rightly… “would the young people be sufficiently equipped and have sufficient skills?” There was a thought that it might take the research down a different path that we didn't particularly want. So from my memory, the discussions were quite pointed really, with a couple of people really selling the merits of involving young people and then I would say it was probably born from there and has grown ever since. (Adult Researcher [AR]4: 27‐35)

Questions centred on if, when and how young people could contribute within a “scientific” framework. Bringing together established demands of academic work with emergent practices of involvement “breached” the implicit expectations of the researchers.^39^ People with prior involvement experience within the team helped others to understand different forms that involvement could take and facilitated necessary “leaps of faith.” An early example of this emerged when UP Group members were asked to comment on the existing design of a certificate to be given to teenage participants. The involvement lead suggested they create a new certificate, as a tangible and manageable first task.

The young people were going to design a certificate and there was great anxiety about what that certificate was going to come back looking like and credit to everybody, they let the young people run with it and actually it was quite conservative (laughs) *…* you could see the learning coming from that … trusting the young people. (AR3: 41‐47)

Further into the research, as tasks became more complex, the involvement facilitators found other ways to harness the creative opportunities arising from differences in expectations.

I think the way we managed to approach it was by inviting members of the management group to some of our early sessions to help them understand that it had to be very, very interactive, it had to be very task orientated and it wasn't about standing in front of a room and telling the young people what was happening… I think there were still tensions … and it was definitely a power imbalance ‐ as a management team we need to get this work done and it's perhaps not getting done as quickly as we hoped. (AR8: 92‐107)

The work of managing hopes and doubts was not just central in the initial phase but was returned to over time. Values and attitudes appeared to shift steadily as the work occurred. The ideas of those that initially championed involvement became part of the way the group began to think. As an adult researcher outlined, through “experiencing the difference and value of what young people bring in,” a transformation occurred. They noted that:

[S]ome of the team members, in the anxieties they had about young people being involved, or being given responsibility, or whatever, well that's in the past because the project team has grown with it, and has learnt from it(AR3: 28‐31)

However, this was not about a key moment of “conversion” but rather a process of learning over time. An involvement facilitator described an element of the cyclical process:

Little cycles all the time where you think… “we've achieved collaboration”… back to… “we are being consulted again” (AR8: 281‐283)

Enabling collaborative working was not just about selecting appropriate methods but also about accommodating hopes, expectations and anxieties, not least of the young people. In the focus groups, they articulated hopes around improving services for other users, as well as developing their own skills. Similarly to the adult researchers, they recalled having doubts in the early stages but to do with the challenges of meeting new people and being in a group. For example, one young person noted how “I was doubtful I could last the two hours (of the first meeting) to be honest” ((UP Group) member [UP]1; Focus Group [FG]1: 24).

Most of the young people had experience of accessing health‐care services for a range of physical, developmental and emotional conditions and were not used to formal meetings and large groups. They were encouraged to bring a trusted adult with them, and some brought a parent, a learning mentor from school, or sibling. Within 2‐3 meetings, most chose to come on their own. Periodically, mini “task and finish” cycles via email were attempted between meetings, but the young people did not engage with this. They soon came to value the sociability of the face‐to‐face meetings. In informal discussions over time, many considered that the consistency of both the young people and the facilitation staff was central in their ability to become adept at managing in the group.

The involvement facilitators hoped for time early on, for group formation and upskilling of the young people. Their doubts and anxieties focused on the interface between the adult researchers and the young people and managing their relationships with both groups.

[We] were very, very clear what active involvement was and it wasn't about changing the hearts and minds of the management team, because they truly believed they needed to actively involve the young people. They probably just hadn't used such an embedded approach… in the past and I think it often challenged some of their beliefs and values around how much empowerment to give young people. (AR8: 205‐210)

A further comment from a young person reflected upon the challenge of accommodating differences and using them as reflective mechanisms for exploring new ways of working.

Researchers see the end point, without thinking about all the ways to get there. Young people see all the possible ways forward, without necessarily keeping the end point in mind. You need both! (UP 2; FG4: 48‐50)

To support such a coming together, the facilitators required experience of involvement methods and an ability to effectively mediate between stakeholders, to accommodate the different ways of thinking but also the emotional and social processes that played out.

### 3.2 Negotiating the work

Interactions and negotiations between and within groups were central. For example, to accommodate the need for the young people's group to form, as well as starting first research tasks, the PSWs were recruited, as the UP Group was set up to provide some early input to research tasks. Initially, the involvement facilitators reported thinking that:

“Let us just let the group form; think what we want in the role of the peer support workers; the added benefits to the research‐… what identity did the group want?; how did they want to be represented?” So within 5 or 6 sessions, I think everybody had almost found their identity and role in that group. (AR8: 178‐182)

During this early period, the involvement lead was prioritizing initial tasks with the adult researchers and “commissioning briefs” were identified as a useful way to communicate and begin working together.

The involvement facilitators reflected on this iterative process in their team discussions, feeling they had to demonstrate, at different times, degrees of resilience (eg to be able to constructively challenge and assimilate various views); sensitivity (eg to convey outcomes that did not meet with initial expectations); negotiation skills (eg to manage points of power, responsibility and decision making); reflexivity (ie to move between representing the young people's views and expressing their own opinions); and pragmatism (eg balancing the desire for a priori conceptual alignment with getting on with the work of task delivery). They were aware of the importance of their debriefing, reflective discussions as a team, to enable them in this work.

It was very much about keeping a strong foundation about the core beliefs around involvement, but actually being pragmatic as to how we were going to deliver within the timescales the (adult researcher) management group needed. (AR8: 118‐121)

The young people reported that they felt they were given an overall aim and structure but with the freedom to work within that. Data from the focus groups suggest they embraced this, often recalling their research experiences with excitement and a sense of fun. Their reflections suggest they experienced less anxiety when considering possible ways of being involved, compared to the adult researchers. The adult researchers had questions about whether young people could engage with a research process maybe because they were already immersed within pre‐defined structures of (adult) academia. The young people experienced less doubt, maybe because they were not embedded within any given system in relation to their new role.

I think researchers often struggle with stripping themselves bare because they've gone through a journey of fighting for funding, fighting for a role, fighting for evidence based literature, fighting for their place in research, proving something or adding value … and then involvement strips all that back again, because you have to leave yourself open and transparent. (AR8: 355‐362)

The adult researchers reflected upon the impact upon themselves of experiencing such a new way of doing things.

### 3.3 Witnessing involvement work

One of the first tasks of the young people was to contribute to the programme's launch event. This was a visible role at an early stage. They appeared to have made a lasting impression and, importantly, set a foundation for their further involvement. For example, one researcher outlined how their expectations were breached.

I thought “the [adult] researchers will say what we're doing, and then somebody important will say something, and somebody else will say something, and then the young people will say something almost as an afterthought.” That's just a reflection of my poor thinking at the time. Actually putting them [the young people] up first was just terrific, it just set the tone. (AR5: 157‐163)

In this way, we see a shift from involvement work rendered as potentially tokenistic practice to being positioned as something that framed the direction of that event.

Beyond the launch, contact between adult researchers and young people was often mediated by a range of proxy actors. The young people were always able to decide how they took tasks forward, often delivering on things in a different way than might have been anticipated by the adults. They also had a work stream that they led on, around the scope and utility of health passports as a tool for young people using health‐care systems. They were supported in this latter work by the involvement facilitators with little direct input from the research team. All aspects of the young people's work were outlined at the formal research management meetings, by the involvement lead as a proxy in the early years and then increasingly, with young people attending themselves. This was a space that attempted to build an overview and coordinate the diverse elements of the whole research programme and was attended by the senior researchers.

A junior researcher also acted as a proxy when they met with the young people to discuss a key data collection tool. The young people's input was seen as central to shaping the tool, and this was reported back to the adult researchers. The young people decided to use the work they had done on this tool, to develop an interactive learning resource for professionals, which has been made available via one of the voluntary organization partners of the research programme. This demonstrates the flexibility that was required to support young people not just with the tasks that were more predictable but with the unexpected opportunities and the added impacts that can emerge.

Times when adult researchers witnessed, either first‐hand or through proxies, the input of the young people seemed to be critical steps in the reflexive development of the involvement work, when relatively invisible work became visible to them. For example, three young people acting as the PSWs were also proxies for UP Group members in the first months. An adult researcher noted:

[T]hat for me was the single thing that was most powerful, a couple of young people [Peer Support Workers] being able to articulate their own views and the views of others at a very practical level, talking about how the young people felt and what the young people said they would be able to do. (AR4: 49‐53)

The PSWs reflected that at such times, they seemed able to “surprise” the adult researchers, as they demonstrated the value of the young people's work.

However, this transformation in perception seems to have occurred even more powerfully when the adult researchers engaged with the UP Group members directly and not via proxies. They routinely remarked on how the young people delivered beyond their expectations. They outlined how they demonstrated ideals like “professionalism” and “objectivity.”

I just didn't really know to what extent we'd get real insights from the young people and so I think I've had my eyes opened wide about a lot of things. This last meeting we attended when they were presenting the results of their initial consultations about health passports … it was a really skilled piece of (I.T.) programming …it was just admirable to see what they'd produced. (AR5: 175‐185)

Having a proxy, some form of mediator, likely helps to manage anxieties and the complexity of interactions required for active involvement. However, when the groups did meet in person, bringing different systems together was manageable.

We shouldn't be having things done to young people …we do need to listen and hear and value their experience and that definitely needs to inform both what we ask ‐[the] questions ‐ [and] how we interpret the findings. …We do have to impose structure and rigor and all those kind of things but unless we have this live experience and the interactions, I think it's our loss. (AR9: 619‐624)

The young people also highlighted the importance of face‐to‐face interactions. When asked if they thought the adult researchers appreciated their input, one young person noted that “Yes, they make the effort whenever they see us to make us feel that way” (UP 3; FG4: 68‐69). Looking to the final stages of the programme, they described wanting further opportunities for directly working together. One young person outlined that:

We would like the managers to come and tell us about their work, or make a short video for us, like we did for them, to see at one of our meetings. (UP 4; FG4: 83‐85)

Even once involvement processes are occurring and working well, it remains important to be aware of different stakeholder views and the need for continued, two‐way dialogue.

### 3.4 Appraising value

In witnessing young people's input, the adult researchers appeared to place an increasing value on it, particularly on a sense of the “authentic voice” conferred upon the research. They highlighted that young people offered a “real‐life” view, with a multidimensional narrative being achieved: “The whole is greater than the parts. By their angle coming in as well, it makes the whole thing much more interesting and relevant” (AR1: 469‐471). They noted how the young people could offer a clarity and directness of message. They reflected on the evolving nature of the process and the importance of not taking a mechanistic approach, with overdefined, a priori goals.

There's a lot more trust and faith in [the young people] than I think people would've imagined possible …and whatever our ambitions at the start, we succeeded. Whether or not it looks like what it was meant to look like (pause) but I think that's fine with public engagement … I think we should have that more emergent agenda, rather than a “we will do this and then we will do this.” (AR6: 677‐671)

In this way, their vision of how “good” involvement could and should be organized was transformed away from a more consultative approach. They all felt that they would engage with the process again, with a renewed sense of the possibilities. They also stressed that such work should be led by those with appropriate skills and experiences in involvement work. The focus on the more emergent nature of the work was also echoed by the involvement facilitators.

There are lots of examples of good practice. To get it right every time is really, really challenging ‐ … [We] could spend the next 10 years coming up with a fantastic tool kit… but actually, depending on the nature of the research, the method of the research and the individuals, their role and development, there is not a one size fits all model. (AR8: 456‐463)

Our findings informed the work within the programme moving into the final year. More opportunities for face‐to‐face working were sought and discussions about the potential for young people's input, despite questions as to how this might be achieved, happened.

Now, they don't necessarily have that skill to stand up in front of people and tell them the key results the research programme. Maybe they do maybe they don't. But if they don't, we have an obligation to, if they're willing to do it, train them to do so. … Supporting them in writing pieces for publications, or being involved in media opportunities. Why shouldn't they be the ones to actually to get the message across to young people? But they can't do that in isolation, they need some sort of support from us. (AR7: 369‐373)

Three of the young people did take key roles in the formal, national dissemination event at the close of the programme, including presenting the results of their own work streams and being on the interactive expert panel. They reflected upon the event, considering that they could not have undertaken such roles when they started the work and one said they felt proud of the way in which “*experts and politicians*” had valued their views. It is clear that building upon the positive experiences across the whole research team, cycles of understanding, behaviour and value formation continued to occur through the course of the programme.

## 4 DISCUSSION

In this study, a need for cyclical, dynamic and flexible approaches to involvement working is suggested. The face‐to‐face work of building relationships is highlighted, along with the need to focus on the emotional, as well as the practical issues that arise. We suggest that this is likely to be relevant to all involvement work, but a central challenge is to understand how approaches might need to be adapted when working with young people in research, as distinct from younger children and adults.

Acknowledging and working with difference was a central finding in this study. Differences were presented as questions and doubts: for example, Will young people be scientific enough? Will I be able to take part in meetings? and Will we be able to effectively bring together adult researchers and young people? The questions had an emotional resonance, with concerns that differences might derail or block processes rather than enhance them. Participants described potential ways to acknowledge and navigate the challenges and stated a desire to not resort to potentially tokenistic ways forward. They demonstrated an understanding that avoiding doubts and difference, no matter how appealing a way forward this seemed at times, was not likely to be helpful.

Exploring the utility of critical discourse in social science, Burman^40^ argues that by assuming universalities and overlooking differences, rather than exploring ambiguity and variety, we reach a position of “banality” with seemingly shared, yet narrow understandings. Similarly, Cook^41^ acknowledges the importance of “messiness” in research:

In research, having multiple view‐points, where each new view and theory is a springboard for further reflection, is an important way of finding new ways of seeing.

Our findings suggest that in order to achieve such discourse, continued reflexive appraisal is required to realize potential difficulties and respond with creative solutions. The current lack of involvement evidence means that issues specific to a project and other local factors are likely to be as important in defining possible ways forward. In this study, descriptions of ways to acknowledge difference and promote discourse included roles for mediators and proxies; central tasks for involvement facilitators; the PSW role; and using commissioning briefs and other ways to support “leaps of faith.” Other mechanisms have been described in studies of adult involvement^12^, ^42^ and the emerging young people's involvement literature.^16^, ^29^

In this way, instrumental involvement actions and guidance are being proposed,^7^, ^8^, ^42^, ^43^ but we also need to consider the processes within which these actions need to be enabled. Current involvement practice can be seen as occurring within transformative “liminal spaces” where fundamental contradictions can arise, requiring communicative rather than instrumental action.^13^ An overly prescriptive use of guidance to pre‐specify the structure of involvement work should be guarded against, as it does not show us how to manage all various challenges and emergent opportunities that involvement working presents,^44^ especially when working with young people.

We might look to agile models of working^45^ adopted in technology and other industries, where an awareness of local problems without a known solution, and the potential skills needed, quickly brings people with varied expertise together, to work within sprint cycles. There is an openness to acknowledge that some elements of the work might succeed and some might not. Any learning is taken forward into the next sprint cycle. Such a cyclical pattern was described within our findings, and the involvement facilitators seemed to describe their requirement for an agile, reflexive skill set. Fox^46^ also describes agility when involving young people in research:

Spaces which are constantly shifting, where young people can change decisions … disrupt power relationships and simultaneously challenge the traditional practices of detailed research plans made months in advance.

The openness and transparency required for such agile and liminal ways of working is articulated in this study, with a suggestion that academics need to let go of aspects of the “professionalized self.” It is suggested that involvement work requires a critically curious standpoint, rather than being fixed on maintaining supposed existing positions that are likely to be based on assumptions and generalizations.^40^ They are also likely to be based upon pre‐existing power relationships, as described in an examination of how experiential capital gained by patients might be recognized alongside the academic capital held by researchers.^47^ Face‐to‐face working is suggested in this study as a key mechanism for exploring the “liminal knowledge spaces” between teenagers and adults, and between service users and academics. Constructivist approaches to adult learning^48^ stress the importance of recurring experiential opportunities, and not just knowledge acquisition, in the on‐going transformation of thinking and practice. Similarly, the learning of children and young people is reported as a social and cyclical process, based on co‐operation and interdependence, with face‐to‐face working as a key mechanism.^49^ Within our own study, we have seen how direct social and verbal exchanges facilitated deeper levels of understanding and the mutual negotiation of meaning. We need to come together to negotiate a balance between: “ill‐informed social experiments where any [involvement] practice is legitimate… [and] …the determinism of top‐down control by experts”.^50^ However, it is suggested that currently, no easy consensus will be reached:

Both literature and practice remain mired in a ‘conceptual muddle’…and the principles underlying the why, whom and how of (involvement) remain confusing and contradictory.^49^

To strengthen the debate, we need to move beyond a focus on proving the worth of involvement outputs, and consider involvement as a complex social process.^13^, ^51^, ^52^, ^53^, ^54^

## 5 CONCLUSION

Adult service users may have a range of work‐based skills or experience of other structures that will shape their approach to a research role, yet questions would likely still arise about their research “credentials” in the liminal space currently occupied by involvement work. In our study context, the “burden of proof” seemingly needed to justify the efforts of meaningfully involving young people in research may have been heightened by them being aged 15‐25, by perceptions about their lack of professional and work‐based experience. This may have also added to initial anxieties and the sense of “surprise” when adult researchers witnessed the young people's work. We suggest that the findings of this study add a valuable insight into work with young people in research and that adult academics might need to reappraise their assumptions about the capabilities of young people as researchers.

## CONFLICTS OF INTEREST

The authors declare no conflicts of interest.

## ETHICAL CONSENT

The Proportionate Review Sub‐committee of the NRES Committee Yorkshire & The Humber—Leeds East Research Ethics Committee (reference 15/YH/0098).
